# CCAAT/enhancer‐binding protein β overexpression alleviates myocardial remodelling by regulating angiotensin‐converting enzyme‐2 expression in diabetes

**DOI:** 10.1111/jcmm.13406

**Published:** 2017-12-21

**Authors:** Yuanyuan Tie, Chungang Zhai, Ya Zhang, Xiaoteng Qin, Fangpu Yu, Hongxuan Li, MeiRong Shan, Cheng Zhang

**Affiliations:** ^1^ Key Laboratory of Cardiovascular Remodeling and Function Research Chinese Ministry of Education and Chinese Ministry of Health Qilu Hospital of Shandong University Jinan Shandong China

**Keywords:** diabetic cardiomyopathy, C/EBPβ, ACE2, fibrosis, apoptosis

## Abstract

Diabetic cardiomyopathy, a major cardiac complication, contributes to heart remodelling and heart failure. Our previous study discovered that CCAAT/enhancer‐binding protein β (C/EBPβ), a transcription factor that belongs to a family of basic leucine zipper transcription factors, interacts with the angiotensin‐converting enzyme 2 (ACE2) promoter sequence in other disease models. Here, we aimed to determine the role of C/EBPβ in diabetes and whether ACE2 expression is regulated by C/EBPβ. A type 1 diabetic mouse model was generated by an intraperitoneal injection of streptozotocin. Diabetic mice were injected with a lentivirus expressing either C/EBPβ or sh‐C/EBPβ or treated with valsartan after 12 weeks to observe the effects of C/EBPβ. *In vitro*, cardiac fibroblasts and cardiomyocytes were treated with high glucose (HG) to investigate the anti‐fibrosis, anti‐apoptosis and regulatory mechanisms of C/EBPβ. C/EBPβ expression was down‐regulated in diabetic mice and HG‐induced cardiac neonatal cells. C/EBPβ overexpression significantly attenuated collagen deposition and cardiomyocyte apoptosis by up‐regulating ACE2 expression. The molecular mechanism involved the binding of C/EBPβ to the ACE2 promoter sequence. Although valsartan, a classic angiotensin receptor blocker, relieved diabetic complications, the up‐regulation of ACE2 expression by C/EBPβ overexpression may exert greater beneficial effects on patients with diabetic cardiomyopathy.

## Introduction

Diabetic cardiomyopathy (DCM), which is characterized by left ventricular (LV) dilatation and systolic dysfunction, occurs independently of recognized causes, such as coronary artery disease, valve disease, arterial hypertension or other cardiovascular diseases 1, 2, 3. LV remodelling is one of the major pathological mechanisms that ultimately lead to congestive heart failure. Numerous mechanisms are involved in the formation and development of LV remodelling in patients with DCM, including myocardial fibrosis, cardiac hypertrophy, mitochondrial damage, inflammatory, apoptosis and activation of the renin‐angiotensin system (RAS) 4.

Homeostasis of the RAS is based on a balance between the angiotensin‐converting enzyme (ACE)–angiotensin (Ang) II–angiotensin II receptor type 1 (AT1R) axis and the ACE2–Ang(1–7)–Ang1–7 receptor (MasR) axis 5. ACE2, a homologue of ACE, has been shown to exert anti‐fibrosis and anti‐hypertrophy effects and to reduce LV remodelling in patients with type 1 diabetes 6, 7. ACE2 catalyses the cleavage of Ang II to Ang(1–7) and then counteracts endogenous ACE by activating MasR 8, 9. Based on the results of clinical trials and experimental studies, the activation of the RAS is associated with the development of LV remodelling in patients with DCM 10, 11. However, the exact mechanism between RAS and DCM remains poorly understood.

CCAAT/enhancer‐binding protein β (C/EBPβ), a transcription factor that belongs to a family of basic leucine zipper transcription factors, affects cell growth and differentiation 12, 13. Reduced C/EBPβ expression up‐regulates the expression of a GATA‐binding protein 4, T‐box transcription factor 5 (Tbx5), NK 2 homeobox 5 (Nkx2.5), α‐myosin heavy chain (α‐MHC), troponin I (TnI) and troponin T (TnT), all of which are hypertrophy‐related genes 14. According to our previous study, C/EBPβ binds the ACE2 promoter sequence and decreases ACE2 expression in Ang II‐treated cells, indicating that C/EBPβ might also regulate ACE2 expression in DCM. We suggested that C/EBPβ overexpression may attenuate collagen accumulation, apoptosis and LV remodelling by regulating ACE2 synthesis and other RAS members in the mouse model of type 1 diabetes.

## Materials and methods

### Animal protocol

All animal experiments were conducted in accordance with the National Institutes of Health guidelines on the care and use of laboratory animals. The protocol was approved by the Animal Care and Use Committee of Shandong University Qilu Hospital. After 1 week of acclimation, 8‐week‐old male C57BL mice (Beijing Hua Fu Kang Biological Polytron Technologies Inc, Beijing, China) were randomized into the control group (*n* = 20) and the treatment group (*n* = 80). Type 1 diabetes was induced in the treatment group by the intraperitoneal injection of 50 mg/kg streptozotocin (STZ; Sigma‐Aldrich, St. Louis, MO, USA) for five consecutive days. Meanwhile, the mice in the control group received intraperitoneal injections of a solvent (0.1 mol/l sodium citrate, pH 4.5). Random blood glucose measurements greater than 16.7 mmol/l in the treatment group for 3 days indicated the successful induction of type 1 diabetes (ACCU‐CHEK Active; Roche, Indianapolis, IN, USA). After 12 weeks, the diabetic mice were randomized into the following four groups: (*i*) DM + shRNA negative control (N.C.) (*n* = 20), (*ii*) DM + C/EBPβ (*n* = 20), (*iii*) DM + sh‐C/EBPβ (*n* = 20) and (*iv*) DM + valsartan (*n* = 20). Mice in the indicated groups were injected with 1 × 10^7^ UT/30 μl of lentivector containing sh‐N.C., C/EBPβ or sh‐C/EBPβ (GENECHEM, Shanghai, China) through the caudal vein. Valsartan (30 mg/kg; Novartis, Beijing, China) dissolved in normal saline was administered by gavage to the mice in the valsartan group 15. Sixteen weeks after the first STZ injection, all the mice were killed.

### Echocardiography

The heart function and dimension parameters were measured using a standard protocol after 16 weeks by transthoracic parasternal echocardiography using the VEVO770 imaging system (VisualSonics, Toronto, ON, Canada). LV parameters, including the left ventricular end‐diastolic diameter (LVEDd), left ventricular posterior wall thickness (LVPWd), left ventricular ejection fraction (LVEF) and fractional shortening (FS), were measured in M‐mode *via* the long/short axis view. The ratio of the early peak (E, mm/sec.) to the late peak (A, mm/sec.) mitral flow velocities was determined using pulsed‐wave Doppler echocardiography.

### Histology and immunohistochemistry

After fixation with 4% paraformaldehyde, dehydration with an alcohol gradient and embedding in paraffin, the heart tissues were cut into 4.5 μm sections. Sections were stained with haematoxylin and eosin (H&E) to measure the cardiomyocyte width and with Masson's trichrome to assess the collagen content. Immunohistochemical staining was performed on sections using a previously described method 16. Sections were incubated with the following primary antibodies at the appropriate concentrations overnight at 4°C: anti‐C/EBPβ, anti‐ACE2, anti‐ACE, anti‐transforming growth factor‐β1 (TGF‐β1), anti‐collagen I and anti‐collagen III (all from Abcam, Cambridge, MA, USA). The secondary antibodies were used according to the manufacturer's specifications. Images of the LV sections were obtained at 400× magnification and measured using the computer software ImagePro Plus 6.0.2 (Media Cybernetics, Houston, TX, USA).

### ELISA of myocardium and blood serum

Ang II and Ang(1–7) levels in myocardium were measured according to the standard protocols (Jianglaibio, Shanghai, China). Blood serum from anaesthetized mice was harvested to measure the serum contents of interleukin‐6 (IL‐6), monocyte chemoattractant protein‐1 (MCP‐1), matrix metalloproteinase (MMP)‐2, MMP‐9 (all from R&D Systems, Quantikine ELISA, Minneapolis, MN, USA), Ang II and Ang(1–7) (Bioswamp, Shanghai, China).

### Cell culture

Primary neonatal cardiac fibroblasts (CFs) were isolated from 1‐ to 3‐day‐old C57 mice according to a previously described protocol 17. The CFs and H9C2 cardiomyocytes were cultured in DMEM (10% foetal bovine serum) in 5% CO_2_ and 95% humidified air at 37°C. Fibroblasts or cardiomyocytes were randomly divided into six groups and exposed to the following different treatments: (*i*) control (5.5 mM glucose), (*ii*) high mannose (osmotic control, oc), 5.5 mM glucose + 27.5 mM mannose), (*iii*) high glucose + sh‐N.C. (HG + sh‐N.C., 33.3 mM glucose), (*iv*) HG + lentivirus vector containing C/EBPβ (HG + C/EBPβ), (*v*) HG + lentivirus vector containing the C/EBPβ‐shRNA (HG + sh‐C/EBPβ) and (*vi*) HG + valsartan (10^−6^ mol/l; Sigma‐Aldrich). Cells were infected with the lentivirus for 24 hrs and then cultured with high glucose for 48 hrs. The valsartan treatment was conducted for 24 hrs. After 72 hrs, cell culture supernatants were collected to measure the activities of MMP‐2 and MMP‐9 using gelatin zymography (GENMED, Shanghai, China), as previously described 17. CFs were randomly selected for treatment with (*i*) sh‐N.C. + si‐N.C., (*ii*) si‐N.C. + C/EBPβ and (*iii*) C/EBPβ + siRNA‐ACE2 (GenePharma, Shanghai, China) for approximately 72 hrs.

### Western blot

Western blots were performed using previously described methods 18. The blots were incubated with the following specific primary antibodies overnight at 4°C on a shaker: anti‐C/EBPβ (Abcam, Cambridge, MA, USA and Santa Cruz Biotechnology, Santa Cruz, CA, USA), anti‐ACE2, anti‐ACE, anti‐AT1R, anti‐AT2R, anti‐TGFβ1, anti‐MMP2, anti‐MMP9, anti‐collagen I, anti‐collagen III (all from Abcam), and B‐cell lymphoma/leukaemia‐2 (Bcl‐2), anti‐Bcl2‐associated X protein (Bax) and anti‐MasR (all Cell Signalling Technology, Boston, MA, USA). All protein levels were normalized to β‐tubulin (Proteintech Group, Wuhan, Hubei, China). Bands were detected using an Amersham Imager 600 (Fairfield, CT, USA) and were quantified with Photoshop CS6 (San Jose, CA, USA).

### Chromatin immunoprecipitation assay

Chromatin immunoprecipitation (ChIP) was performed using the SimpleChIP Enzymatic Chromatin IP Kit (#9003) obtained from Cell Signaling Technology (Boston, MA, USA) according to the standard protocol. Immunoprecipitated DNA was harvested from HG‐induced HeLa cells using an anti‐C/EBPβ antibody (Santa Cruz Biotechnology), normal rabbit IgG and an anti‐histone 3 antibody. ChIP‐enriched DNA was amplified using standard PCR methods and the following primer sets: ACE2: 5′‐ACCGGTTTTGATTTGGCCAT‐3′ (sense) and 5′‐CAGTAAACAATCTGCTGAGCCA‐3′ (anti‐sense).

### Statistical analysis

All data from at least three independent experiments were expressed as mean ± SD, and intergroup differences were analysed using a one‐way anova *via* SPSS software 18.0 (SPSS, Chicago, IL, USA). *P* < 0.05 was regarded as statistically significant.

## Results

### Fasting blood glucose concentrations and morphometric profiles

As expected, 1 week after STZ injection, fasting blood glucose concentrations in diabetic mice showed a marked elevation that persisted until the end of the experiment (Table 1). Excessive water intake, excessive food intake and polyuria were observed in the diabetic mice, particularly in the DM + sh‐N.C. and DM + sh‐C/EBPβ groups. Meanwhile, differences in body weight, heart weight and the ratio of heart weight to body weight were statistically significant among the five groups (Table 1). Thus, C/EBPβ overexpression might reverse cardiac remodelling.

**Table 1:** Characteristics of the five groups of mice after 16 weeks of treatment

Parameters	Control	Diabetic mice			
		DM + sh‐N.C.	DM + C/EBPβ	DM + sh‐C/EBPβ	DM + valsartan
Blood glucose (mmol/l)	8.32 ± 0.23	24.12 ± 0.35^a^	23.90 ± 0.33^a^	23.97 ± 0.24^a^	23.70 ± 0.35^a^
Body weight (BW, g)	27.83 ± 0.89	24.13 ± 1.03^a^	24.47 ± 0.46^a^	24.09 ± 1.47^a^	23.74 ± 0.78^a^
Heart weight (HW, mg)	120.54 ± 4.32	145.58 ± 3.17^a^	129.19 ± 3.23^a^^,^ b	146.57 ± 2.33^a^	135.38 ± 2.47^a^^,^ b ^,^ c
HW/BW (mg/g)	4.33 ± 0.17	6.04 ± 0.28^a^	5.27 ± 0.33^a^^,^ b	6.10 ± 0.28^a^	5.66 ± 0.07^a^^,^ b ^,^ c

a *P* < 0.05 *versus* control.

b *P* < 0.05 *versus* DM + sh‐N.C.

c *P* < 0.05 *versus* DM + C/EBPβ.

### C/EBPβ overexpression and the valsartan treatment ameliorated myocardial remodelling

**Figure 1:**
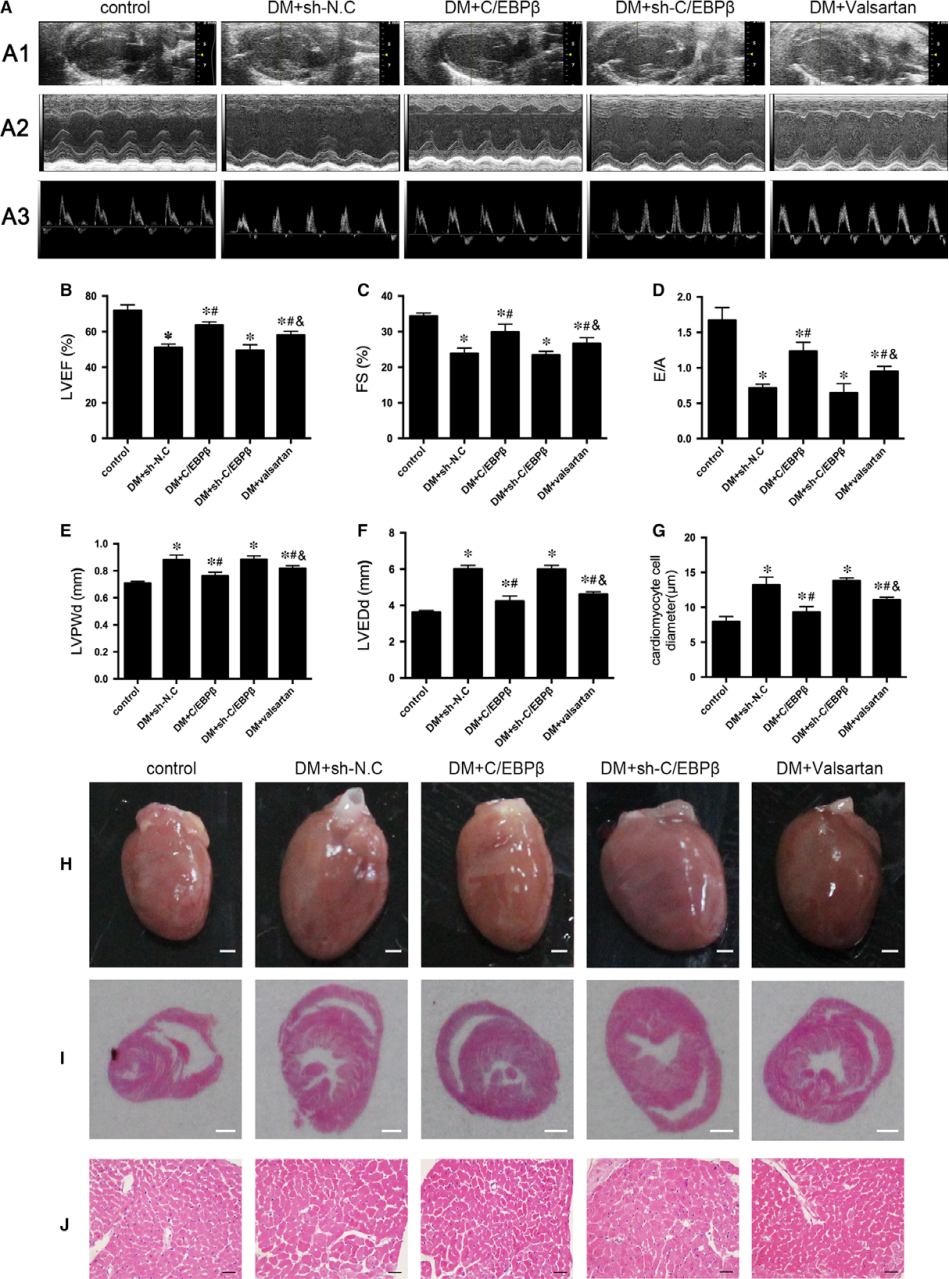
Functional and pathological features of hearts. (**A1** and **A2**) Representative two‐dimensional echocardiograms and M‐mode echocardiograms. (**A3**) Representative pulsed‐wave Doppler echocardiograms of mitral inflow. (**B–F**) Sequential statistics results: LVEF, FS, E/A, LVPWd and LVEDd. (**G**) Quantitative analysis of cardiomyocyte cell diameter. (**H**) Heart size (scale bar: 3 mm). (**I**) Representative cross‐sectional image of histological staining illustrating the anatomy at the papillary muscle level (scale bar: 3 mm). (**J**) Representative images of H&E‐stained sections (scale bar: 20 μm). Control: normal mice. DM + sh‐N.C.: negative shRNA control in diabetic mice. DM + C/EBPβ: lentivirus‐mediated delivery of C/EBPβ in diabetic mice. DM + sh‐C/EBPβ: lentivirus‐mediated delivery of sh‐C/EBPβ in diabetic mice. DM + valsartan: valsartan treatment in diabetic mice. Data are presented as the means ± SD. *P < 0.05 *versus* control, #*P* < 0.05 *versus* DM + sh‐N.C., &*P* < 0.05 *versus* DM + C/EBPβ.

Echocardiography was employed to evaluate cardiac function at the end of the experiment. LVEF, FS and the E/A ratio were substantially decreased, and LVEDd and LVPWd were increased in the DM + sh‐N.C. group compared with those in the controls. Compared with the DM + sh‐N.C. group, the DM + C/EBPβ and DM + valsartan groups exhibited improvements in LVEF, FS and the E/A ratio and decreases in LVEDd and LVPWd, but the DM + sh‐C/EBPβ group was not significantly different from the DM + sh‐N.C. group (*P* < 0.05; Fig. 1A–F). The increased heart size and cardiomyocyte width were reduced by C/EBPβ overexpression and valsartan (*P* < 0.05; Fig. 1G–J). Although the valsartan treatment improved cardiac function indices and attenuated heart size and cardiomyocyte width, C/EBPβ overexpression had a much better effect on myocardial remodelling.

### C/EBPβ overexpression ameliorates extracellular matrix deposition *in vitro* and *in vivo*

**Figure 2:**
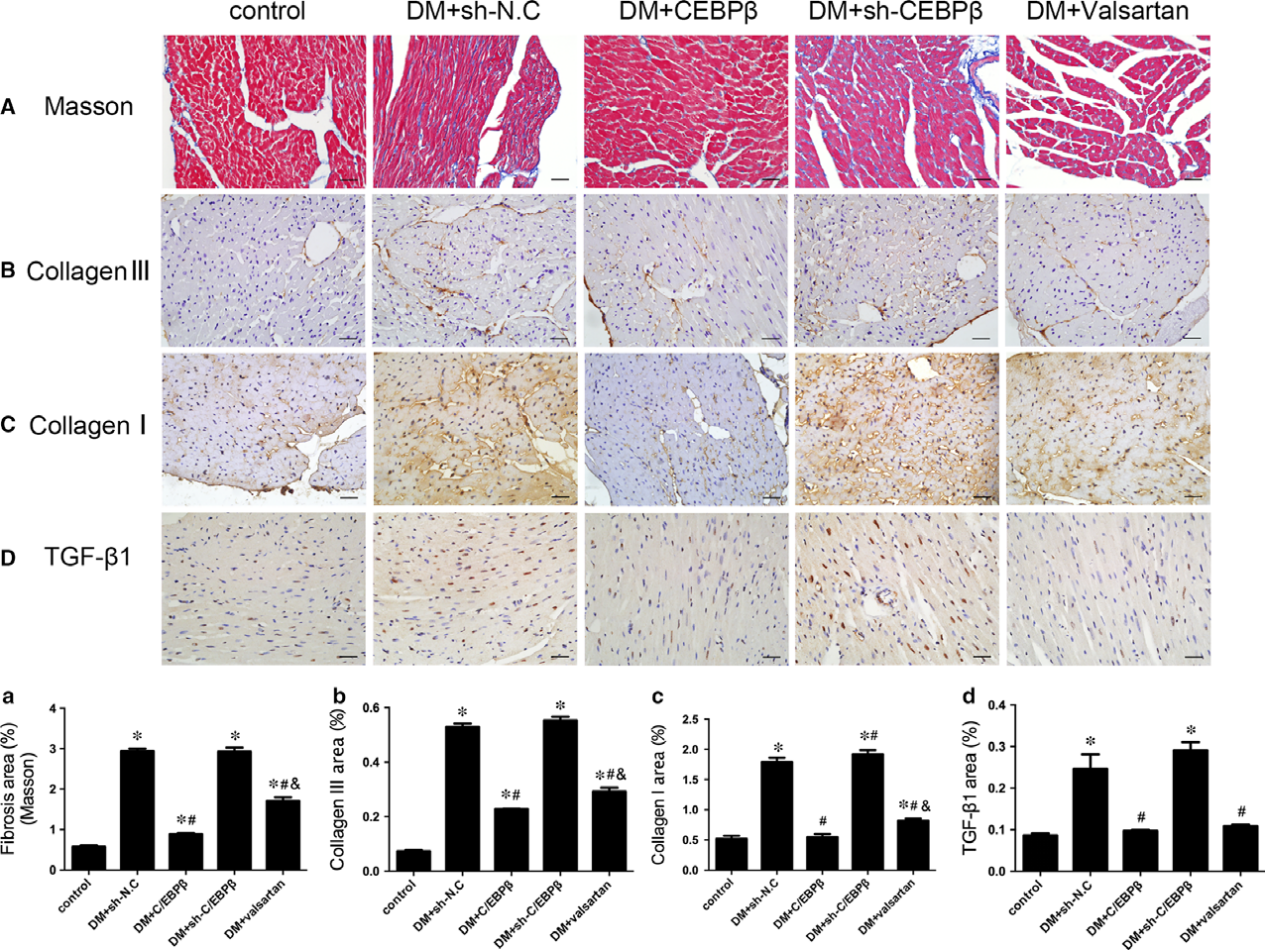
Histological and immunohistochemical analyses of myocardial fibrosis. (**A**) Masson's trichrome staining (first row; scale bar: 20 μm). (**B**–**D**) Immunohistochemical staining and quantitative analysis of collagen III (**B**, second row; scale bar: 20 μm), collagen I (**C**, third row; scale bar: 20 μm) and TGF‐β1 expression (**D**, fourth row; scale bar: 20 μm). Data are presented as the means ± SD. *P < 0.05 *versus* control; #*P* < 0.05 *versus* DM + sh‐N.C.; &*P* < 0.05 *versus* DM + C/EBPβ.

Masson's trichome staining of cardiac sections revealed the elevated expression of the extracellular matrix (ECM) in the interstitial areas of diabetic mice compared to the expression in the control mice. C/EBPβ overexpression and the valsartan treatment dramatically reduced collagen deposition in the intramyocardial and perivascular regions compared to diabetic mice that were transfected with sh‐N.C. Additionally, the expression was much lower in the C/EBPβ overexpression group than in the valsartan group. The DM + sh‐C/EBPβ group was not significantly different from the DM + sh‐N.C. group (*P* < 0.05; Fig. 2A).

**Figure 3:**
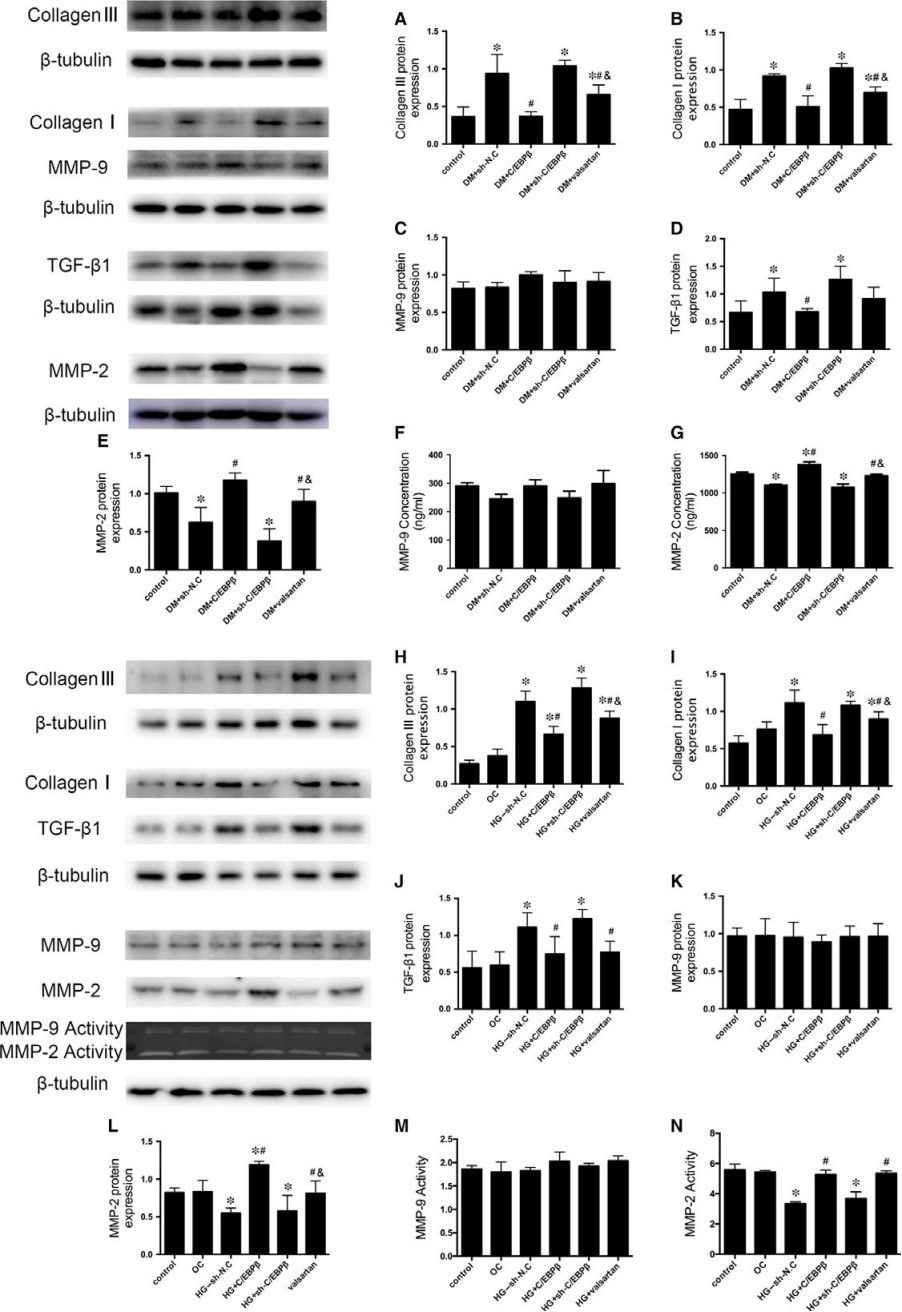
Effects of C/EBPβ on collagen deposition and TGF‐β1, MMP‐2 and MMP‐9 expression *in vivo* and *in vitro*. Serum MMP‐2 and MMP‐9 levels were measured using enzyme‐linked immunosorbent assay (ELISA). (**A–E**) Sequential Western blot evaluations of collagen III (**A**), collagen I (**B**), MMP‐9 (**C**), TGF‐β1 (**D**) and MMP‐2 (**E**) levels in the myocardium. (**F–G**) ELISA analysis of serum MMP‐9 and MMP‐2 levels. (**H–L**) Sequential Western blot evaluations of collagen III (**H**), collagen I (**I**), TGF‐β1 (**J**), MMP‐9 (**K**) and MMP‐2 (**L**) levels in primary neonatal mouse CFs. (**M** and **N**) MMP‐9 and MMP‐2 activities in cell culture supernatants were assessed using zymography. Control: 5.5 mM glucose. oc: osmotic control, 5.5 mM glucose + 27.5 mM mannose. HG + sh‐N.C.: high glucose (33.3 mM glucose) + negative shRNA control. HG + C/EBPβ: high glucose + lentivirus vector containing C/EBPβ. HG + sh‐C/EBPβ: high glucose + lentivirus vector containing shRNA‐C/EBPβ. HG + valsartan: high glucose + valsartan (10^−6^ mol/l). Data are presented as the means ± SD. *P < 0.05 *versus* control; #*P* < 0.05 *versus* DM + sh‐N.C. or HG + sh‐N.C.; &*P* < 0.05 *versus* DM + C/EBPβ or HG + C/EBPβ.

The induction of diabetes increased the accumulation of the fibrotic markers collagen I, collagen III and TGF‐β1 compared to that in healthy controls. C/EBPβ overexpression and the valsartan treatment reduced the levels of collagen and TGF‐β1 compared to those in the DM + sh‐N.C. group, and collagen was expressed at much lower levels in the C/EBPβ overexpression group than in the valsartan group. The DM + sh‐C/EBPβ group was not significantly different from the DM + sh‐N.C. group. The effects of all groups were confirmed by immunohistochemistry and Western blotting (*P* < 0.05; Figs 2B,C and D and 3A,B and D).

According to the Western blot results, MMP‐9 expression was not significantly different from all groups (*P* < 0.05; Fig. 3C). But C/EBPβ overexpression and the valsartan treatment ameliorated the diabetes‐induced reduction in MMP‐2 levels, and MMP‐2 was expressed at much higher levels in the C/EBPβ overexpression group than in the valsartan group. The DM + sh‐C/EBPβ group was not significantly different from the DM + sh‐N.C. group (*P* < 0.05; Fig. 3E). Consistent with the Western blot results, the ELISA showed that the serum MMP‐9 levels were not significantly different from all groups, whereas C/EBPβ overexpression and the valsartan treatment increased the serum MMP‐2 levels compared to those in the DM + sh‐N.C. group, and MMP‐2 levels were much higher in the C/EBPβ overexpression group than in the valsartan group. The sh‐C/EBPβ treatment decreased MMP‐2 levels, but the difference was not significantly different from the DM + sh‐N.C. group (*P* < 0.05; Fig. 3F and G).

Consistent with the alterations observed in diabetic mice, the expression of the collagen I and III and TGF‐β1 proteins was increased in high glucose‐treated CFs. Both C/EBPβ overexpression and the valsartan treatment attenuated the up‐regulation of the collagen I and III and TGF‐β1 compared to expression in the HG + sh‐N.C. group, and collagen was expressed at lower levels in the C/EBPβ overexpression group than in the valsartan‐treated group. The HG + sh‐C/EBPβ group was not significantly different from the HG + sh‐N.C. group (*P* < 0.05; Fig. 3H–J). Likewise, the results of MMPs proteins expression in tissue were verified by exposing CFs to HG (*P* < 0.05; Fig. 3K and L). Additionally, significant differences in activity of MMP‐9 were not observed among the groups, but C/EBPβ overexpression and the valsartan treatment both remarkably ameliorated the HG‐induced decrease in MMP‐2 activity (*P* < 0.05; Fig. 3M and N).

### C/EBPβ overexpression suppresses apoptosis in the diabetic myocardium and H9C2 cardiomyocytes and decreases inflammatory factors expression in serum

**Figure 4:**
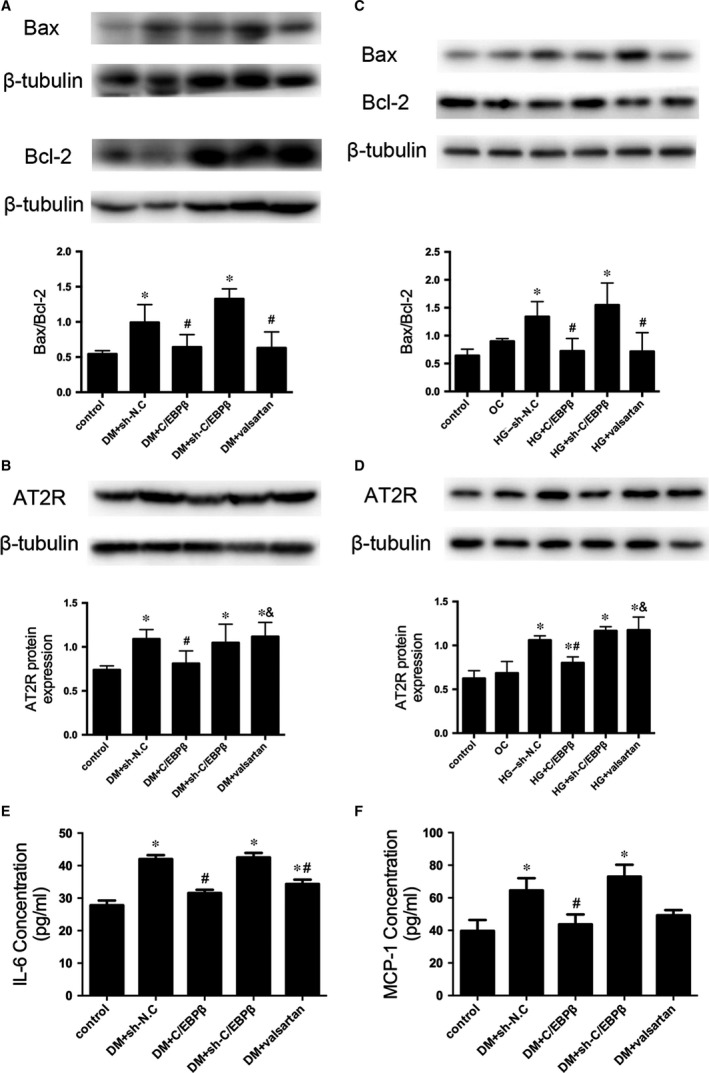
Effects of C/EBPβ on apoptosis and inflammation. (**A**) Western blot analysis of Bax and Bcl‐2 expression in the myocardium. (**B**) Western blot analysis of AT2R expression *in vivo*. (**C**) Bax and Bcl‐2 protein expression in H9C2 cells. (**D**) Western blot analysis of AT2R expression in H9C2 cells. (**E–F**) Serum interleukin‐6 (IL‐6) and monocyte chemoattractant protein‐1 (MCP‐1) levels were measured using ELISAs. Data are presented as the means ± SD. *P < 0.05 *versus* control; #*P* < 0.05 *versus* DM + sh‐N.C. or HG + sh‐N.C.; &*P* < 0.05 *versus* DM + C/EBPβ or HG + C/EBPβ.

The Bax/Bcl‐2 ratio and AT2R expression were increased in vehicle‐treated diabetic mice compared with expression in the healthy controls. C/EBPβ overexpression effectively reduced the Bax/Bcl‐2 ratio and AT2R expression compared to that in the DM + sh‐N.C. group, whereas the valsartan treatment only exerted a significant effect on the Bax/Bcl‐2 ratio. The apoptosis indices of the DM + sh‐C/EBPβ group were not significantly different from that of the DM + sh‐N.C. group (*P* < 0.05; Fig. 4A and B). The results were verified by exposing H9C2 cardiomyocytes to HG (*P* < 0.05; Fig. 4C and D).

Serum IL‐6 and MCP‐1 levels were increased in the diabetic mice, but they were alleviated by C/EBPβ overexpression and the valsartan treatment compared to the levels in the DM + sh‐N.C. group. The DM + sh‐C/EBPβ group was not significantly different from the DM + sh‐N.C. group (*P* < 0.05; Fig. 4E and F).

### Diabetes and the HG treatment decrease C/EBPβ expression, and C/EBPβ overexpression up‐regulates ACE2 expression

**Figure 5:**
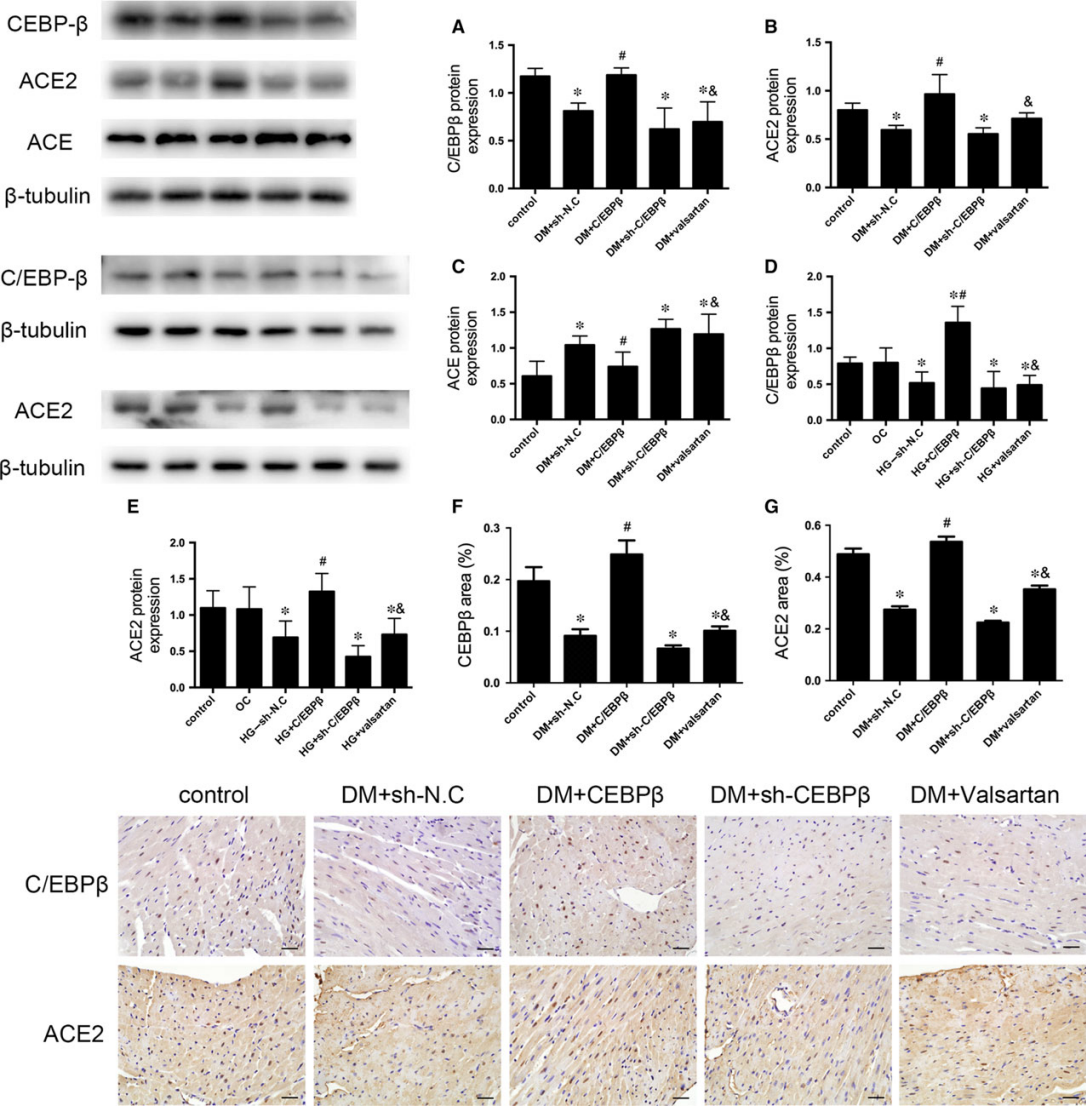
C/EBPβ and ACE2 protein expression levels *in vivo* and *in vitro*. ACE protein expression level in the myocardium. Western blot analysis of the levels of the C/EBPβ (**A**) and ACE2 (**B**) ACE (**C**) proteins in mouse heart tissue. Levels of C/EBPβ (**D**) and ACE2 (**E**) proteins in CFs. Immunohistochemical analysis of myocardial C/EBPβ (**F**) and ACE2 (**G**) expression. Data are presented as the means ± SD. *P < 0.05 *versus* control; #*P* < 0.05 *versus* DM + sh‐N.C. or HG + sh‐N.C.; &*P* < 0.05 *versus* DM + C/EBPβ or HG + C/EBPβ.

The C/EBPβ protein was expressed at lower levels in the sh‐N.C. group than in the controls, but it was up‐regulated in the C/EBPβ overexpression group. The sh‐C/EBPβ and valsartan‐treated groups showed no significant differences compared with the vehicle‐treated group (*P* < 0.05; Fig. 5A). Meanwhile, the ACE2 protein was expressed at lower levels in the sh‐N.C. group than in the control group. C/EBPβ overexpression increased ACE2 protein expression compared to that with the sh‐N.C. treatment. The sh‐C/EBPβ and valsartan‐treated groups were not significantly different from the vehicle group *in vivo* (*P* < 0.05; Fig. 5B). Similar effects of C/EBPβ and ACE2 expressions were verified in CFs treated with HG and in the myocardium by immunohistochemistry (*P* < 0.05; Fig. 5D–G).

### C/EBPβ overexpression decreases Ang II and increases Ang(1–7) levels in DCM

**Figure 6:**
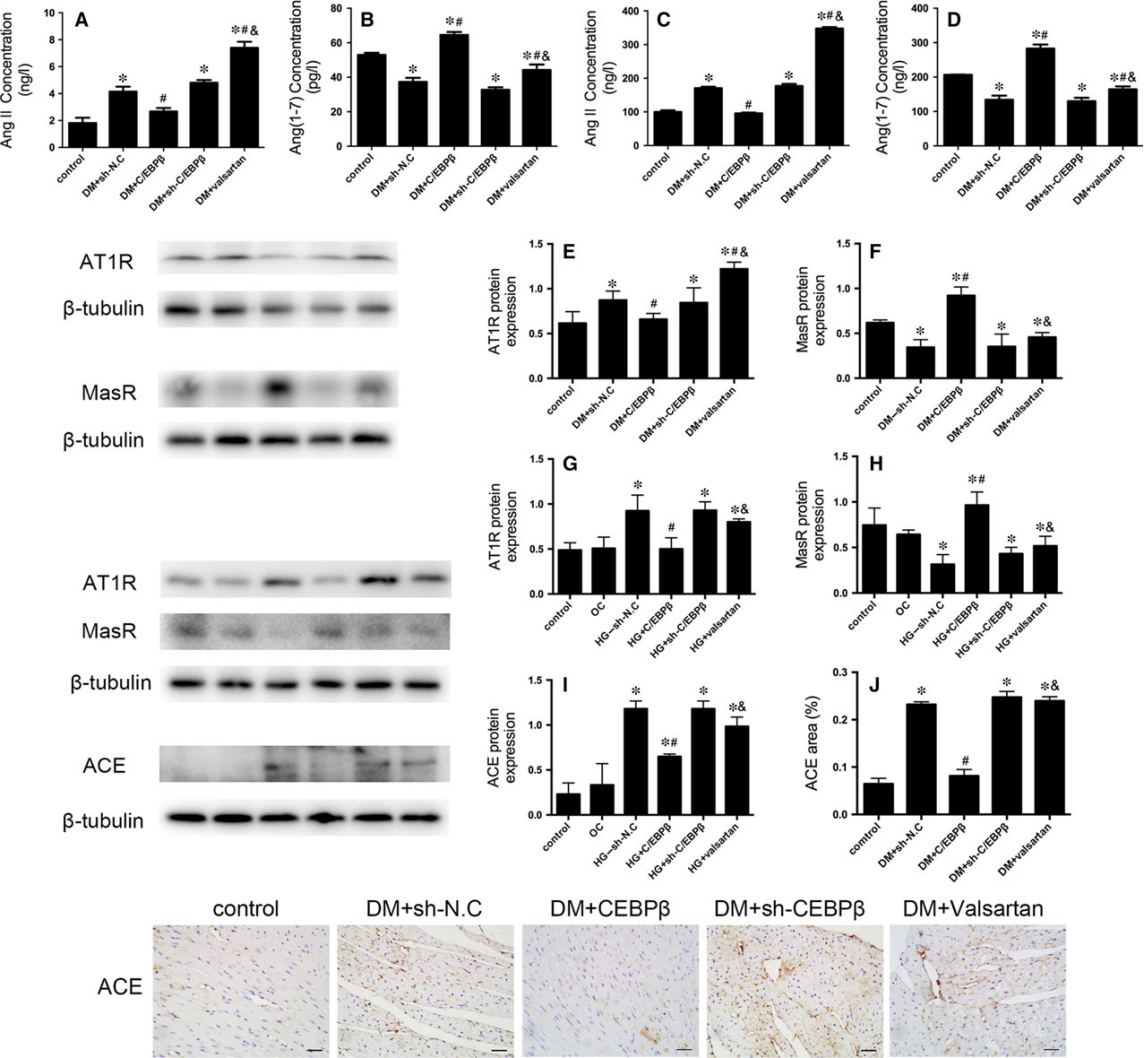
Ang II and Ang(1–7) levels in the myocardium and serum were measured using ELISAs. Western blots of the levels of the ACE, AT1R and MasR proteins *in vivo* and *in vitro*. Immunohistochemical analysis of myocardial ACE expression. (**A–D**) Ang II and Ang(1–7) levels in the myocardium (**A** and **B**) and serum (**C** and **D**) were measured using ELISAs. (**E** and **F**) Western blot analysis of the levels of the AT1R and MasR proteins in mouse heart tissue. (**G** and **H**) Levels of the AT1R and MasR proteins in CFs. (**I** and **J**) Western blots and immunohistochemical staining for ACE in CFs and in the myocardium (scale bar: 20 μm). Data are presented as the means ± SD. *P < 0.05 *versus* control; #*P* < 0.05 *versus* DM + sh‐N.C.; &*P* < 0.05 *versus* DM + C/EBPβ.

The sh‐N.C. treatment, C/EBPβ silencing and valsartan treatment markedly increased myocardium Ang II levels in diabetic mice compared to the levels in the controls. C/EBPβ overexpression alleviated the increased Ang II content compared to the sh‐N.C. treatment. The levels of Ang (1–7) were significantly increased in the C/EBPβ overexpression group and slightly increased in the valsartan‐treated groups compared to the sh‐N.C. group. The sh‐N.C. treatment and C/EBPβ silencing decreased the myocardium Ang(1–7) levels, but the difference was not statistically significant (*P* < 0.05; Fig. 6A and B). Serum Ang II and Ang(1–7) levels are consistent with those in tissue (*P* < 0.05; Fig. 6C and D).

### Levels of the ACE, AT1R and MasR proteins in the diabetic myocardium and CFs

Diabetes significantly elevated the expression of the ACE and AT1R proteins and decreased the expression of the MasR protein. C/EBPβ overexpression remarkably reduced levels of the ACE and AT1R proteins and increased levels of the MasR protein, but the valsartan and C/EBPβ silenced groups were not significantly different from the sh‐N.C. group (*P* < 0.05; Fig. 6E and F). The results were verified in HG‐treated CFs (*P* < 0.05; Fig. 6G and H). The expression of ACE was tested consistently by Western blot *in vivo* and *in vitro* and verified by immunohistochemistry analysis (*P* < 0.05; Figs 5C and 6I and J).

### C/EBPβ overexpression alleviates fibrosis and apoptosis by up‐regulating ACE2 levels

**Figure 7:**
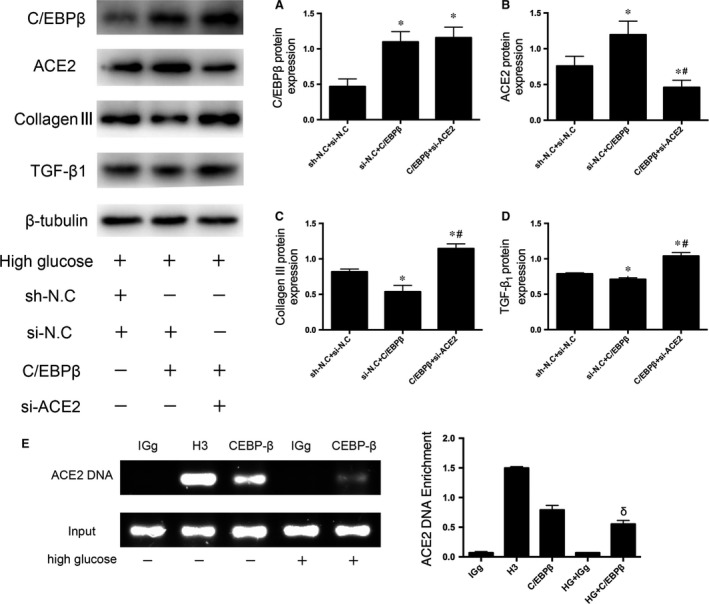
Mechanisms by which C/EBPβ and ACE2 respond to the HG treatment in CFs. Cells were divided into sh‐N.C. + si‐N.C., si‐N.C. + C/EBPβ and C/EBPβ + si‐ACE2 groups. (**A–D**) Western blot analysis of C/EBPβ (**A**), ACE2 (**B**), collagen III (**C**) and TGF‐β1 (**D**) levels. HG: high glucose, 33.3 mM glucose. sh‐N.C. + si‐N.C.: shRNA negative control + siRNA negative control. si‐N.C. + C/EBPβ: siRNA negative control + lentivirus‐mediated delivery of C/EBPβ. C/EBPβ + si‐ACE2: lentivirus‐mediated delivery of C/EBPβ + siRNA‐ACE2. (**E**) Chromatin immunoprecipitation (ChIP) analysis of the relationship between C/EBPβ and ACE2. IgG: negative control. H3: positive control. C/EBPβ: C/EBPβ antibody. HG + IgG: high glucose + negative control. HG + C/EBPβ: high glucose + C/EBPβ antibody. Data are presented as the means ± SD. *P < 0.05 *versus* sh‐N.C. + si‐N.C.; #*P* < 0.05 *versus* si‐N.C. + C/EBPβ. δ < 0.05 *versus* C/EBPβ.

Consistent with the changes described above, ACE2 was expressed at significantly higher levels in CFs in the si‐N.C + C/EBPβ group than in the sh‐N.C. + si‐N.C. group and decreased in the C/EBPβ + si‐ACE2 group compared to the levels in the si‐N.C. + C/EBPβ group. Additionally, the expression of collagen III and TGF‐β1 was not reduced in the C/EBPβ + si‐ACE2 group compared to expression in the si‐N.C + C/EBPβ group, which corroborated our hypothesis regarding the C/EBPβ‐ACE2 pathway (*P* < 0.05; Fig. 7A–D).

ChIP assays were used to obtain further insights into the molecular interactions between C/EBPβ and ACE2 following the HG treatment. The ACE2 promoter sequence was obviously enriched in the DNA immunoprecipitated with the anti‐C/EBPβ antibody, indicating that C/EBPβ directly binds to the ACE2 promoter. Furthermore, the results verified that the binding of C/EBPβ to the ACE2 promoter decreased following the HG treatment, further indicating that hyperglycaemia‐induced ACE2 down‐regulation may be directly caused by the weaker binding force between C/EBPβ and the ACE2 promoter (*P* < 0.05; Fig. 7E). Based on these findings, C/EBPβ regulates ACE2 expression by directly binding to its promoter.

## Discussion

The major finding in our study was that C/EBPβ overexpression ameliorated diabetes‐induced myocardial remodelling by up‐regulating ACE2 expression and modulating the expression of other members of the RAS.

The most important effect of C/EBPβ on the successful model was the amelioration of DCM‐induced fibrosis and remodelling. Enhanced fibrosis in cardiac tissue is a vital hallmark of cardiac dysfunction. Ang II, which is converted from Ang I by ACE, is one of the most important factors that contribute to the up‐regulation of collagen expression in patients with diabetes 19, 20. Ang II stimulates collagen synthesis in fibroblasts and myofibroblasts *via* AT1R 21. Candesartan inhibited the increased collagen I expression in HG‐treated fibroblasts in a previous study, indicating that Ang II regulates HG‐induced collagen deposition in CFs 22. The up‐regulation of ACE2 and Ang(1–7) expression alleviates the fibrosis and cardiac dysfunction in subjects with DCM 7, 23. In the present study, C/EBPβ overexpression decreased Ang II levels and reduced collagen production and ECM deposition in HG‐treated fibroblasts and diabetic mice. As shown in our previous study, diabetic mice and CFs both expressed increased levels of TGF‐β1, which acts as a major mediator of cardiac remodelling by altering collagen metabolism and inducing cardiomyocyte hypertrophy 24, 25. TGF‐β1 expression is induced by Ang II in cardiac cells 26, 27. Ang II inhibition has been reported to reduce ECM synthesis by modulating TGF‐β1 expression in CFs and diabetic rats 28, 29. In the present study, levels of the TGF‐β1 protein were significantly suppressed by C/EBPβ overexpression or the valsartan treatment, whereas diabetes and sh‐C/EBPβ exacerbated fibrosis *in vitro* and *in vivo* by up‐regulating TGF‐β1 expression, indicating that C/EBPβ overexpression reduces ECM deposition by decreasing Ang II‐induced TGF‐β1 expression.

An imbalance between ECM deposition and degradation in the heart plays a major role in fibrosis in DCM. The expression and activity of MMP‐2 mediate collagen degradation and are down‐regulated in diabetic mice and in CFs treated with HG or Ang II 7, 25, 30, 31. ACE2 overexpression reduces ECM deposition by increasing MMP‐2 activity and expression 7. MMP‐2 primarily degrades collagen, which consists of fibrillary peptides and newly generated fibres. However, MMP‐9, which is similar to MMP‐2, also degrades collagen, although with lower proteolytic activity 25. The sh‐N.C. and sh‐C/EBPβ treatment enhanced the reduced MMP‐2 levels, whereas C/EBPβ overexpression increased MMP‐2 expression and activity by up‐regulating ACE2 expression, thus increasing the degradation of the ECM in mice with DCM. However, MMP‐9 activity was not significantly altered among the groups in our study.

An increase in cardiomyocyte loss and hypertrophy underlies cardiac fibrosis and dysfunction in diabetes 32. As expected, the Bax/Bcl‐2 ratio, which reflects the rate of apoptosis, was higher in diabetic mice and in HG‐induced cardiomyocytes, consistent with previous findings 18. Ang II triggers apoptosis and exacerbates cell growth, both of which are vital pathological features in DCM 33, 34. AT2R, which has controversial functions, was expressed at high levels in the diabetic mice and HG‐induced CFs in our study. However, according to some studies, AT2R, like MasR, opposes AT1R activity and counteracts the negative effects induced by AT1R 35. An increase in AT2R levels accompanied by a reduction in Bcl‐2 levels was previously observed in DCM. Specific stimulation of AT2R after serum starvation exacerbates apoptosis 36. In addition, AT2R exerts a proapoptotic effect on neonatal cardiomyocytes and R3T3 mouse fibroblasts 37. Based on our results, AT2R promoted apoptosis in diabetes. C/EBPβ overexpression not only decreased the Bax/Bcl‐2 ratio but also reduced AT2R expression in DCM. Although the exact mechanism by which C/EBPβ regulated AT2R was not investigated in the present study, our findings suggest a new method for studying apoptosis in DCM.

Based on our study and previous research, the mechanisms by which C/EBPβ alleviated fibrosis and apoptosis were mainly due to the up‐regulation of ACE2 expression and reduction of Ang II levels. In a previous study, ACE2 was recognized as an important modulator of DCM 38. However, as an enzyme, the development of ACE2 as a clinical drug is very difficult due to its instability and ease of degradation. Therefore, future studies should focus on identifying a factor that induces ACE2 expression or activity. Using ChIP assays, we revealed a new regulatory mechanism in which C/EBPβ binds to the ACE2 promoter sequence and enhances ACE2 production as a transcription factor. The HG treatment reduced the expression of C/EBPβ and its binding to the ACE2 promoter. Meanwhile, the expression of fibrosis markers was not reduced following treatment with C/EBPβ + ACE2‐siRNA. Therefore, following up‐regulation by C/EBPβ, ACE2 catalyses the cleavage of Ang II to Ang(1–7) and inhibits ACE function. Ang(1–7) alleviates fibrosis and cardiac dysfunction by activating MasR, leading to reductions in ACE, AT1R and AT2R levels, as verified in previous studies 7, 23. However, we did not comprehensively examine whether the effects of C/EBPβ suppression on fibrosis and apoptosis in DCM depended on ACE2 completely, and the new mechanism by which C/EBPβ regulates DCM warrants further investigation.

Valsartan and other ARBs have been shown to prevent DCM. We also believe that the effects of valsartan and other ARBs on relieving DM are undeniable. However, ARBs have been shown to relieve diabetic complications by specifically blocking rather than circulating intracellular Ang II. Furthermore, intracellular Ang II is more relevant to fibroblasts in diabetic hearts, and several research studies have indicated that some effects of intracellular Ang II are not inhibited by ARBs 7, 39. In our study, C/EBPβ up‐regulated ACE2 expression, ACE2 catalysed the cleavage of Ang II to Ang(1–7) and decreased intracellular Ang II level and counteracted the effects of ACE on CFs and cardiomyocytes in ameliorating diabetic fibrosis and cardiac dysfunction, but valsartan had no effect on ACE2 expression 7. As AT1R antagonists, valsartan only effects on AT1R and has no effect on AT2R and MasR according to its pharmacological action. C/EBPβ up‐regulated Ang(1–7), which decreased ACE, AT1R and AT2R levels, and up‐regulated MasR expression levels 7, 23, which ultimately prevented DCM progression, whereas valsartan increased Ang(1–7) levels much lower than C/EBPβ. Overall, C/EBPβ decreased intracellular Ang II level by up‐regulating ACE2 and Ang(1–7) levels, indicating that C/EBPβ may be more effective in treating DCM than ARBs.

In conclusion, we first investigated the role of C/EBPβ as a transcription factor that promotes ACE2 expression and C/EBPβ overexpression as a protective factor against fibrosis and apoptosis by up‐regulating ACE2 expression in DCM. Additionally, the underlying mechanisms involve the down‐regulation of the ACE–Ang II–AT1R axis and the up‐regulation of the ACE2–Ang(1–7)–MasR axis that directly suppressed collagen deposition and apoptosis. Furthermore, C/EBPβ‐induced ACE2 up‐regulation is more efficacious in treating DCM than ARBs. Our study revealed a novel potential therapeutic target for the amelioration of cardiac dysfunction and remodelling in patients with DCM.

## Conflict of interests

The authors confirm that there are no conflicts of interest.

## Supporting information


**Figure S1** Fluorescent protein expression levels in myocardium by immunofluorescence technique. The left was the control group treated with a streptozotocin solvent and the right received negative shRNA treatment.
