# Supplementary material for: CCAAT/enhancer‐binding protein β overexpression alleviates myocardial remodelling by regulating angiotensin‐converting enzyme‐2 expression in diabetes
Source: J Cell Mol Med. 2017 Dec 21;22(3):1475–88. doi: 10.1111/jcmm.13406 (PMC5824391; doi:10.1111/jcmm.13406)
Supplement: Supplementary file 2 [file JCMM-22-1475-s002.docx]

Figure legend for S1

Fluorescent protein expression levels in myocardium by immunofluorescence technique. The left was the control group treated with a streptozotocin solvent and the right received negative shRNA treatment.
